# Reliability of timed walking tests and temporo-spatial gait parameters in youths with neurological gait disorders

**DOI:** 10.1186/s12883-016-0538-y

**Published:** 2016-01-31

**Authors:** Judith V. Graser, Claudia Letsch, Hubertus J. A. van Hedel

**Affiliations:** 1 Paediatric Rehab Research Group, Rehabilitation Centre, University Children’s Hospital Zurich, Affoltern am Albis, Switzerland; 2 Children’s Research Centre, University Children’s Hospital Zurich, Zurich, Switzerland; 3 Neuroscience Centre Zurich (ZNZ), Zurich, Switzerland; 4 Institute of Human Movement Sciences and Sports, ETH Zurich, Zurich, Switzerland

**Keywords:** Gait capacity, 6 Minute walk test, 10 Meter walk test, Paediatric neurorehabilitation, Psychometric properties, GAITRite, Gait symmetry, Child, Cerebral palsy

## Abstract

**Background:**

The 10-Meter Walk Tests (10MWT) and the 6-Minute Walk Test (6MinWT) are applied to assess gait capacity in paediatric patients. To better objectify changes in qualitative aspects of gait, temporo-spatial parameters like stride length or step symmetry could be simultaneously assessed with a GAITRite system. Reliability has not yet been evaluated in a heterogeneous sample of children with various neurological gait disorders such as is representative for paediatric neuro-rehabilitation. The aim of this study was to assess test-retest reliability of the 10MWT, the 6MinWT and simultaneously recorded gait parameters captured with the GAITRite system in children with neurological gait disorders.

**Methods:**

This is a cross-sectional study with two measurement time-points. Thirty participants (9 females; mean (standard deviation) age 13.0 (3.6) years, 10 with cerebral palsy, 6 after stroke, among other diagnoses) performed the 10MWT at preferred (10MWTpref) and maximum speed (10MWTmax) and the 6MinWT on two occasions (mean time interval: 7.0 (1.9) days). Relative reliability was quantified with an intra-class correlation coefficient (ICC); the measurement error reflecting absolute reliability was quantified with the standard error of measurement and the smallest real difference.

**Results:**

ICCs of timed walking tests (time measured with a stopwatch, step count for the 10MWT and walking distance for the 6MinWT) ranged from 0.89–0.97. ICCs of temporo-spatial gait parameters ranged from 0.81–0.95 (10MWTpref), from 0.61–0.90 (10MWTmax) and from 0.88–0.97 (6MinWT). In general, absolute reliability was greatest in the 6MinWT.

**Conclusion:**

Timed walking tests and temporo-spatial gait parameters obtained from the GAITRite system appear reliable in children with neurological gait disorders. However, especially in children with poorer walking ability, the reliability of temporo-spatial parameters might have been positively influenced, as unclear steps had to be removed using the GAITRite software. As absolute reliability is rather low, the responsiveness of these measures needs to be further evaluated.

## Background

Worldwide the prevalence of Cerebral Palsy (CP) varies between 1.5 to 2.5 per 1000 births [1] and has increased over the years [2]. While CP is the most common physical disability among children [3], traumatic brain injury is also a leading cause of long-term disability among children and young adults [4]. Also, children with stroke, myelomeningocele, spinal cord injury, genetic disorders or various developmental disorders are frequently undergoing paediatric neurorehabilitation; therefore, the population in this field is very heterogeneous.

Many children with neurological disorders such as CP, traumatic brain injury, or stroke and their parents prioritise walking above any other activities to be improved during rehabilitation [5]. Therefore, to evaluate the effectiveness of the rehabilitation program, a careful evaluation of walking ability is required. The international classification of functioning, disability and health (ICF) declares walking as an item on the activity and participation domain (WHO, 2001). Therefore, at least part of its assessment should also occur in this domain.

The 10-Meter Walk Test (10MWT) and the 6-Minute Walk Test (6MinWT) have often been used to assess gait capacity in paediatric patients [6–8]. Commonly, the time required to walk 10 m is recorded while the covered distance is measured for the 6MinWT.

Interestingly, while psychometric studies evaluating the reliability of the 10MWT performed at a comfortable speed in children with neurological disorders do not seem to exist, test-retest reliability of the 10MWT performed at fast speed proved to be acceptable [6]. Also, the 6MinWT proved reliable when tested in healthy children [9], in children with myelomeningocele [7] and CP [6, 8]. The test-retest reliability of the 6MinWT was evaluated in a group of 31 children with CP with a retest interval of 1 to 4 weeks [6] and in a sample of 41 children with CP, who performed the test twice on the same day [8]. Bartels et al. examined psychometric properties of the 6MinWT administered in children with chronic paediatric conditions [10]. In their recently published review, they stated that there is little evidence available for responsiveness and measurement error (i.e. absolute reliability).

Combining these tests simultaneously with the GAITRite, which has to our knowledge not been done before, provides additional information on temporo-spatial gait parameters. These parameters allow an objective evaluation of parameters reflecting the quality of gait since, for example, step length or step time symmetry can be computed. Wondra et al. [11] stated that these parameters are often used for the assessment of treatment effects and to expose irregularities in the gait pattern of children with motor disabilities. Several studies showed that most temporo-spatial gait parameters, including e.g. symmetry of steps, were validly and reliably assessed by the GAITRite system in healthy adults [12–14]. In children with developmental coordination disorder, reliability differed considerably between gait parameters [15]. Reliability was found to be satisfying in children with motor disabilities (mostly CP) who walked barefoot or while wearing shoes and orthoses [11] and in 18 children with a mild CP (Gross Motor Function Classification System (GMFCS) level I and II) [16].

Wondra et al. let their participants practice the walk test and measured 6 to 10 trials [11]. This procedure might lead to better reliability since the walks are practiced before the test. Same-day reliability testing is mentioned to be crucial to determine therapeutical interventions with immediate effects, such as when applying an orthosis [11]. In our case, to determine the outcome of a longer intervention period, it makes sense to assess test-retest reliability with an interval that is long enough to allow a certain independence of the measurements, but short enough to ensure that the patient’s performance remains stable.

To obtain reliable pre- and post-measurements it is recommended to have the same person administering both tests. Nevertheless, in the clinic it is often difficult having the same tester performing both pre- and post-assessments. Therefore, determining the reliability between different testers at different time points might reflect better daily routines. In the studies by Wondra et al. [11] and Sohrsdahl et al. [16] there is no information about whether the same person did the tests or whether there was more than one tester.

Even though reliability results obtained in very homogeneous patient groups might make sense for research purposes, their generalizability to the clinics is very limited. The aim of this study was to evaluate the test-retest reliability of the 10MWT performed at preferred speed (10MWTpref), at maximum speed (10MWTmax) and the 6MinWT, including temporo-spatial gait parameters that were simultaneously collected in youths with neurological diagnoses. The first and second test session were performed by different therapists to reflect the normal daily scenario. According to the results presented by Sorsdahl et al. [16] and Wondra et al. [11] and based on our heterogeneous sample that we aimed to include, we hypothesized that most of the parameters would reveal good relative reliability (intra-class correlation coefficients (ICCs) > 0.8). According to our experience with children, we hypothesized that most parameters would show poor absolute reliability, i.e. considerable measurement errors due to high within-subject variance.

## Methods

### Study design

In this cross-sectional study, two different assessors (physiotherapists or movement scientists who had all received the same training) performed two measurements within 14 days maximum (targeted was a between-test interval of 7 days). Since we had to plan the time-points within the regular ongoing rehabilitation program, we choose this pragmatic time range. The rehabilitation program consisted of physiotherapy, occupational therapy, speech and language therapy, school, sports, and/or robot-assisted training, according to the patients’ individual goals.

### Participants

For our convenience sample, we recruited inpatients of the Rehabilitation Centre for Children and Adolescents of the University Children’s Hospital Zurich in Affoltern am Albis, Switzerland. We included youths between the age of 5 and 20 years with gait disorders of neurological origin. Participants had to be able to understand and follow the instructions of the timed walking tests. Excluded were children who were not able to walk without the physical assistance of a person. Walking aids such as walkers as well as orthoses of any kind were allowed. A sample size of 25–30 participants was minimally required since such a sample size was suggested to calculate an accurate estimate of the random error [17].

The Ethics Committee of the Canton of Zurich approved this study. Children below 15 years provided verbal informed consent. Legal representatives and adolescents aged 15 years and older signed a written informed consent.

### Instrumentation

The GAITRite Electronic Walkway Platinum (CIR Systems Inc., Peekskill, N.Y., USA) has an active area of 61 cm × 488 cm containing 18’432 sensors (distance between sensors 1.27 cm). The system captures the geometry and relative arrangement of each footfall as a function of time at a sampling frequency of 120 Hz.

The walkway was placed in a long corridor in a calm environment.

### Procedures

For all timed walk tests, participants wore their regular shoes and orthoses. Participants completed the three timed walking tests in a calm corridor in the order presented below.

#### 10 Meter Walk Test at preferred speed

The time to walk across the 10 middle meters of a 14 m long walkway was recorded (tape on the floor marked the distances) to account for acceleration and deceleration effects [16]. The GAITRite mat was placed at the start of the 10 m walkway.

Participants were instructed the following: “Walk in your normal, comfortable speed up to the last line.” This test was repeated twice, with a short break in between. We recorded the time in seconds and counted the number of steps. The average value of the two measurements was included in the analysis.

#### 10 Meter Walk Test at maximum speed

The 10MWTmax was conducted similarly as the 10MWTpref. Participants were instructed as follows: “Walk as fast as you can to the last line, but so that you are still feeling safe and without running.” The test was also repeated twice, with a short break in between. Only data from the fastest trial were included in the analysis, as this test should indicate the participant’s maximal capacity.

#### 6 Minute Walk Test

**Fig. 1:**
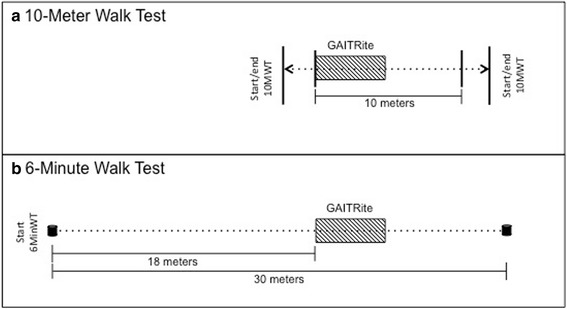
Setup of the (**a**) 10-Meter Walk Test and (**b**) 6-Minute Walk Test. Shown is the position of the GAITRite walkway in relation to the start of the timed walk tests. For the 6-Minute Walk Test, participants walked repetitively over the GAITRite (walking up and down the corridor)

After a break of 5 to 10 min, participants performed the 6MinWT according to guidelines published by the American Thoracic Society [18]. The participants walked up and down 30 m. The GAITRite walkway was positioned 18 m after the start of the track (Fig. 1). Participants repeatedly walked over it.

At each end of the 30 m track, they circumvented a pylon. Instructions and encouragement were standardized throughout the test. “The aim of this test is to walk as far as possible within 6 min. You walk up and down the hallway; every time you reach the end of the hallway you circumvent the pylon and walk back. If you are tired or exhausted, you are allowed to walk slower, to stop or take a break, but try to keep on walking as soon as possible. Remember, the aim is to walk as far as possible.” During the test, verbal encouragement was given every minute, e.g. after one minute: “You are doing well; you have five more minutes to walk,” after two minutes: “Go on like this; you have four more minutes to go,” etc. The child was instructed to lean against the wall when it needed a break and was informed to continue walking when being able to. Participants were informed 15 s before the end of the tests to stand still soon. The maximal distance covered during 6 min was determined and rounded to the next meter.

A trained physiotherapist performed manual muscle testing of the hip extensor and flexor muscles, the ab- and adductors, the internal and external rotators, the knee extensors and –flexors, and the plantar and dorsiflexors of the ankle joint. Testing occurred after the timed walking tests had been performed. The scores were evaluated according to Durfee and Iaizzo [19].

### Data processing

Simultaneously to the timed walk tests and the recording of step count, and time needed to cover 10 meter (10MWT) or distance walked during 6 minutes (6MinWT), the GAITRite system captured the footfalls. These were then processed into various temporo-spatial gait parameters with the accompanying software. Incomplete footfalls, scrub marks and marks produced by walking aids were deleted. Included were only walks with at least 4 footfalls. Data were then exported to Microsoft Excel. For the 10MWTpref, the two trials were averaged, while for the 10MWTmax, data from the fastest trial (measured with a stopwatch) were selected. For the 6MinWT, the average of all the walks crossing the GAITRite was calculated.

We selected the following GAITRite parameters for analysis: velocity, cadence, double support as a percentage of the gait cycle and step length, as the reliability of these parameters was also previously investigated [11, 16]. The parameters normalized velocity (ratio of velocity and leg length), step-extremity-ratio (ratio of leg- and step-length) and step time were chosen since we considered them important for describing a gait pattern. Double support, step length, step-extremity-ratio and step time were determined for the left and right leg and the more and the less affected leg. The more affected leg was defined as the leg with the smaller sum-score of muscle strength scores [20] (Table 1). If the sum-score was the same on both legs, the left leg was classified as more affected. Furthermore, we calculated an absolute symmetry index (ASI) for the step time- and step length-symmetry according to the following formula:

**Table 1:** Participant characteristics

Sex	9 girls, 21 boys	
Mean (SD) age, years	13.0 (3.6)	
Mean (SD) weight, kg	44.9 (16.9)	
Mean (SD) height, cm	151.2 (18.3)	
Values of MMT (Maximum: 45 points/leg)	Points	
Mean (SD) sum score		
Left leg	33.9 (8.5)	
Right leg	33.7 (8.4)	
More affected leg	32.4 (8.2)	
Less affected leg	35.2 (8.4)	
Diagnoses	Participants	Time since injury/diagnosis/surgery
	*n*	Mean (min-max), days
Cerebral palsy	10	
GMFCS level II:	3	
GMFCS level III:	2	
Missing GMFCS level:	5	
Postsurgery: osteotomy of the femur (right side)	1	132
Postsurgery: selective dorsal rhizotomy	1	102
Postsurgery: shortening of ligamenta patellae (both sides)	1	96
Postsurgery: osteotomy of the femur (right side)/tendon lengthening and shortening	1	82/63
Stroke	6	177 (43–305); one congenital
Traumatic brain injury	6	105 (40–309)
Demyelination of CNS	2	54; one congenital
Astrocytome	2	90 (76–103)
Postinfectuous encephalopathy	1	299
Medulla blastoma	1	1351
Transverse myelopathy	1	74
Ataxia, unclear aetiology	1	Congenital
Walking ability	Participants	
	*n*	
Independent walker	23	
Walking aids:		
Walker	4	
Crutches	2	
4 point crutches	1	
Orthoses:		
Ankle-foot orthoses	8	
Insoles	3	
Foot lifter splint (one side)	1	

*MMT* manual muscle test (values 0 to 5 points where 0 is no activation at all and 5 maximal force against resistance. Nine muscles of each leg were tested, therefore the maximum sum-score is 45 points per leg), *GMFCS* Gross Motor Function Classification System, *CNS* central nervous system

*ASI* = *ABS*[(2 × (*X*_*L*_ − *X*_*R*_)/(*X*_*L*_ + *X*_*R*_)) × 100] [21, 22].

Where *XL* refers to the according parameter of the left leg and *XR* to the same parameter of the right leg and *ABS* to the absolute value_._

An absolute symmetry index value of 0 % refers to perfect symmetry [22] while <10 % is considered normal [23]. We considered symmetry important, as it reflected a qualitative aspect of the gait pattern.

### Data analysis

The statistical analyses were conducted with SPSS (IBM SPSS Statistics V. 19.0). To test for systematic differences between session 1 and session 2 for all parameters (e.g. an improvement due to familiarization) we applied a Wilcoxon signed rank test. To assess relative reliability a two-way random model ICC (2,1) based on absolute agreement was applied. The 95 % confidence interval was calculated to specify the precision of the estimates. The ICCs were evaluated according the Munro’s classification for correlation coefficients: 0.00 to 0.25: little if any correlation; 0.26 to 0.49: low correlation; 0.50 to 0.69: moderate correlation; 0.70 to 0.89: high correlation; 0.90 to 1.00: very high correlation [24]. Nunnally and Bernstein [25] state that a reliability coefficient of 0.70 is sufficient in early stages of research, but recommended are values of 0.80 or higher [26].

For absolute reliability the standard error of measurement (SEM) and the smallest real difference (SRD) were calculated according to the following formulas [27]:

$$ SEM=\sqrt{\Big({\sigma}_t^2}+{\sigma}_e^2\Big) $$

$$ SRD=1.96\times \sqrt{2}\times SEM $$

Here, ${\sigma}_t^2$ refers to the variance of the trial and ${\sigma}_e^2$ to the variance of the residual error. The units of SEM and SRD are those of the particular parameter. To be able to compare the absolute reliability measures between various parameters or various tests, the SEM% and SRD% were calculated as $SEM\%=\left(SEM\div \overline{X}\right)\times 100$, and $SRD\%=\left(SRD\div \overline{X}\right)\times 100$ respectively where is the grand mean of the correspondent parameter of session 1 and 2 [28].

Data were presented as means and standard deviations (SD), despite that most data were not normally distributed. Mean and SD values should enable future studies to use these data for, for example, sample size calculations. To determine differences in relative and absolute reliability between the 10MWTpref, 10MWTmax and the 6MinWT, the ICC and the SEM% values of the temporo-spatial gait parameters were compared among the three timed walking tests with a Friedman’s test and consecutive Wilcoxon tests. For the Wilcoxon tests, a Bonferroni correction was applied and α was set at 0.025, as each dataset was included twice.

## Results

Recruited were 35 participants. Due to technical difficulties, data of two participants could not be used, and three participants were incompliant. Demographical and clinical characteristics of the remaining 30 participants are shown in Table 1. Mean time interval (SD) between test and retest was 7.0 (1.8) days (range: 4–12 days).

All participants had complete 10MWTpref and 10MWTmax datasets. Three 6MinWT datasets were excluded: one participant became incompliant during testing while technical problems with the GAITRite resulted in the loss of two datasets and a reduction of 61 cm in active measurement area in 7 datasets (which reduced the number of recorded footfalls by one or two).

**Fig. 2:**
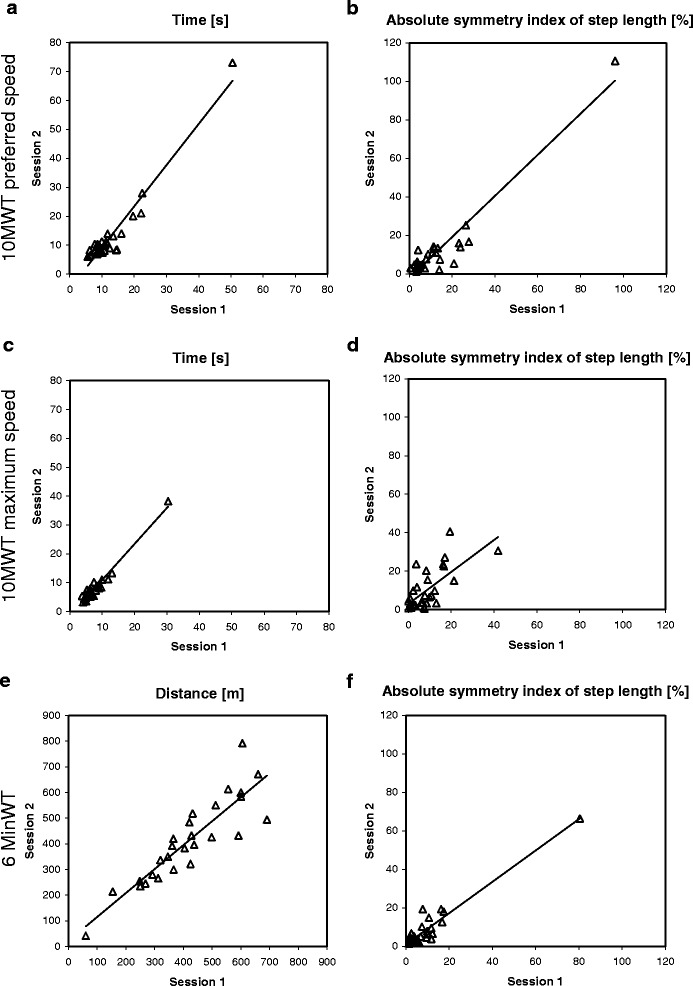
Scatter-plots showing the results of the first and second assessment of the time needed to cover 10 m at (**a**) preferred or (**c**) maximum speed, or (**e**) the distance covered during 6 min. Shown are also the repeated assessments of the absolute symmetry index calculated for the step length for the (**b**) 10-min walking test performed at preferred speed (10MWTpref), (**d**) 10-min walking test performed at maximum speed (10MWTmax) and (**f**) 6-min walking test (6MinWT)

Means, SDs, and results of the Wilcoxon signed rank tests between session 1 and 2 of all the parameters (conventional parameters: stopwatch time, step count, and walking distance and the GAITRite parameters) are displayed in Table 2. Measurement results are also displayed in Fig. 2.

**Table 2:** Means of all the parameters and results of the Wilcoxon signed rank test between session 1 and 2

Parameter	10MWTpref				10MWTmax				6MinWT			
	Session 1	Session 2	Wilcoxon		Session 1	Session 2	Wilcoxon		Session 1	Session 2	Wilcoxon	
	Mean (SD)	Mean (SD)	MD	*p*-value	Mean (SD)	Mean (SD)	MD	*p*-value	Mean (SD)	Mean (SD)	MD	*p*-value
Conventional parameters												
Time (stopwatch) (s)	12.62 (8.35)	12.93 (12.33)	0.26	0.48	7.61 (4.84)	7.95 (6.21)	−0.03	0.76	NA	NA	NA	NA
Step count (n steps)	20.22 (6.41)	20.00 (7.43)	0.00	0.53	16.47 (4.86)	16.80 (5.16)	0.00	0.17	NA	NA	NA	NA
Walking distance (m)	NA	NA	NA	NA	NA	NA	NA	NA	414.93 (155.37)	408.78 (162.96)	0.00	0.69
GAITRite parameters												
Velocity (m/s)	0.97 (0.33)	1.02 (0.34)	−0.04	0.11	1.52 (0.49)	1.51 (0.52)	0.00	0.98	1.26 (0.42)	1.28 (0.47)	0.01	0.92
Normalized velocity (m/s)	1.26 (0.44)	1.34 (0.44)	−0.05	0.09	2.00 (0.74)	1.97 (0.67)	−0.01	0.93	1.66 (0.54)	1.66 (0.57)	0.01	1.00
Cadence (steps/min)	101.72 (20.17)	104.95 (20.65)	−2.13	0.70	133.44 (30.79)	133.83 (29.56)	−3.10	0.80	116.50 (24.04)	116.66 (25.80)	0.64	0.87
Extremity-step-ratio L	0.70 (0.17)	0.73 (0.18)	−0.02	0.06	0.87 (0.19)	0.86 (0.19)	0.03	0.46	0.82 (0.18)	0.82 (0.18)	0.00	0.92
Extremity-step-ratio R	0.75 (0.15)	0.76 (0.15)	−0.02	0.44	0.89 (0.18)	0.88 (0.19)	0.03	0.27	0.84 (0.15)	0.84 (0.16)	−0.01	0.98
Extremity-step-ratio MA	0.70 (0.17)	0.73 (0.18)	−0.02	0.05	0.87 (0.19)	0.87 (0.20)	0.01	0.83	0.82 (0.18)	0.82 (0.19)	0.00	0.79
Extremity-step-ratio LA	0.74 (0.15)	0.76 (0.15)	−0.01	0.55	0.89 (0.18)	0.86 (0.18)	0.04	0.09	0.83 (0.16)	0.83 (0.16)	−0.01	0.83
Double support L (% GC)	30.95 (10.15)	29.89 (10.63)	0.85	0.25	21.97 (7.38)	22.50 (9.19)	−0.50	0.70	26.52 (9.83)	27.13 (11.93)	−0.72	0.40
Double support R (% GC)	31.06 (9.98)	29.74 (10.73)	1.08	0.21	21.88 (7.52)	22.11 (9.08)	0.35	0.55	26.78 (10.46)	27.19 (12.03)	−0.25	0.55
Double support MA (% GC)	30.95 (10.16)	29.95 (10.70)	0.75	0.28	21.90 (7.34)	22.46 (9.22)	−0.20	0.67	26.58 (9.84)	26.99 (11.81)	−0.54	0.65
Double support LA (% GC)	31.06 (9.97)	29.68 (10.66)	1.15	0.18	21.95 (7.57)	22.15 (9.05)	0.60	0.53	26.72 (10.45)	27.33 (12.15)	−0.25	0.37
Step time L (s)	0.59 (0.12)	0.57 (0.11)	0.02	**0.04**	0.46 (0.09)	0.46 (0.12)	0.01	0.90	0.54 (0.15)	0.54 (0.17)	0.01	0.87
Step time R (s)	0.64 (0.24)	0.65 (0.31)	0.02	0.33	0.48 (0.14)	0.49 (0.17)	0.00	0.77	0.57 (0.23)	0.59 (0.33)	−0.01	0.67
Step time MA (s)	0.61 (0.12)	0.59 (0.11)	0.01	0.08	0.47 (0.09)	0.47 (0.11)	0.00	0.71	0.55 (0.14)	0.55 (0.16)	0.00	0.98
Step time LA (s)	0.63 (0.24)	0.63 (0.32)	0.02	0.27	0.48 (0.14)	0.48 (0.18)	0.00	0.80	0.56 (0.24)	0.58 (0.33)	−0.01	0.67
Step time symmetry (%)	12.21 (16.46)	12.48 (16.85)	0.41	0.89	8.91 (8.78)	9.34 (11.69)	0.92	0.81	10.08 (10.18)	9.62 (11.47)	0.29	0.20
Step length L (cm)	53.59 (14.51)	55.94 (15.13)	−1.42	0.06	66.65 (16.20)	65.09 (17.39)	1.30	0.42	61.69 (16.03)	62.92 (16.14)	−0.90	0.20
Step length R (cm)	57.47 (14.41)	58.91 (14.28)	−1.17	0.39	68.66 (16.70)	67.78 (17.92)	2.57	0.45	62.14 (15.42)	64.38 (15.23)	−1.06	0.06
Step length MA (cm)	53.94 (14.52)	56.46 (15.19)	−1.81	**0.04**	67.06 (16.99)	66.13 (18.36)	0.09	0.61	61.91 (16.08)	63.17 (16.22)	−0.57	0.15
Step length LA (cm)	57.12 (14.49)	58.39 (14.30)	−0.92	0.54	68.25 (15.93)	66.74 (17.02)	3.18	0.22	61.92 (15.37)	64.12 (15.16)	−1.24	0.07
Step length symmetry (%)	12.67 (17.52)	11.42 (19.56)	0.79	0.35	8.93 (8.65)	10.33 (10.56)	−0.20	0.52	10.10 (14.86)	9.15 (12.67)	0.71	0.26

Symmetries are displayed in percentages where a value between 0 and 10 % refers to normal symmetry

*p*-value of Wilcoxon signed rank test

bold values refer to significance (*p* < 0.05)

*10MWTpref* 10 meter walk test at preferred speed, *10MWTmax* 10 meter walk test at maximum speed, *6MinWT* 6 min walk tests, *% GC* percentage of the gait cycle, *SD* standard deviation, *MD* median of differences, *L* left leg, *R* right leg, *LA* less affected leg, *MA* more affected leg, *NA* not applicable

Relative reliability varied considerably between the parameters (0.61 < ICC < 0.97) (see also Table 3). ICCs of the 10MWTpref and the 6MinWT met or exceeded the minimum acceptable value of 0.80. 10MWTmax ICC values varied from moderate to very high while 4/23 parameters did not achieve the level of 0.70.

**Table 3:** Relative and absolute reliability measures of all the parameters and walk tests

Parameters	10MWTpref					10MWTmax					6MinWT				
	ICC (95 % CI)	SEM	SRD	SEM%	SRD%	ICC (95 % CI)	SEM	SRD	SEM%	SRD%	ICC (95 % CI)	SEM	SRD	SEM%	SRD%
Conventional parameters															
Time (stopwatch) (s)	0.90 (0.80–0.95)	3.61	10.00	28.26	78.33	0.95 (0.89–0.98)	1.84	5.11	23.69	65.67	NA	NA	NA	NA	NA
Step count (n steps)	0.93 (0.85–0.96)	2.09	5.80	10.40	28.83	0.97 (0.93–0.98)	1.58	4.39	9.52	26.38	NA	NA	NA	NA	NA
Walking distance (m)	NA	NA	NA	NA	NA	NA	NA	NA	NA	NA	0.89 (0.77–0.95)	58.02	160.82	14.09	39.05
GAITRite parameters															
Velocity (m/s)	0.82 (0.66–0.91)	0.26	0.72	26.09	72.25	0.86 (0.73–0.93)	0.20	0.55	13.22	36.36	0.90 (0.80–0.96)	0.14	0.40	11.38	31.55
Normalized velocity (m/s)	0.83 (0.67–0.92)	0.38	1.04	29.28	80.13	0.86 (0.73–0.93)	0.29	0.82	14.59	41.26	0.90 (0.79–0.95)	0.18	0.50	10.83	30.02
Cadence (steps/min)	0.88 (0.76–0.94)	14.20	39.37	13.74	38.10	0.70 (0.45–0.84)	16.92	46.91	12.66	35.10	0.88 (0.75–0.94)	8.93	24.76	7.66	21.24
Extremity-step-ratio L	0.87 (0.74–0.94)	0.13	0.36	18.28	50.62	0.89 (0.78–0.94)	0.08	0.21	9.29	24.39	0.96 (0.92–0.98)	0.04	0.10	4.36	12.09
Extremity-step-ratio R	0.82 (0.66–0.91)	0.09	0.26	11.93	34.46	0.90 (0.81–0.95)	0.09	0.24	10.19	27.16	0.93 (0.86–0.97)	0.04	0.12	5.15	14.29
Extremity-step-ratio MA	0.88 (0.75–0.94)	0.14	0.38	19.53	53.01	0.90 (0.81–0.95)	0.06	0.17	6.91	19.57	0.96 (0.92–0.98)	0.04	0.10	4.51	12.49
Extremity-step-ratio LA	0.81 (0.65–0.91)	0.09	0.25	12.02	33.38	0.89 (0.78–0.95)	0.12	0.32	13.70	36.53	0.94 (0.86–0.97)	0.04	0.11	4.94	13.68
Double support L (% of the gait cycle)	0.84 (0.68–0.92)	5.89	16.34	19.36	53.72	0.75 (0.53–0.87)	4.73	13.11	21.27	58.97	0.93 (0.85–0.97)	3.70	10.26	13.80	38.24
Double support R (% of the gait cycle)	0.82 (0.67–0.91)	6.72	18.61	22.11	61.22	0.74 (0.51–0.86)	4.43	12.29	20.14	55.87	0.95 (0.89–0.87)	2.98	8.26	11.04	30.61
Double support MA (% of the gait cycle)	0.84 (0.68–0.92)	5.75	15.93	18.88	52.32	0.75 (0.53–0.87)	4.76	13.21	21.46	59.55	0.94 (0.87–0.97)	3.15	8.74	11.77	32.63
Double support LA (% of the gait cycle)	0.82 (0.66–0.91)	6.88	19.08	22.65	62.83	0.73 (0.51–0.86)	4.42	12.26	20.05	55.61	0.94 (0.88–0.97)	3.55	9.85	13.15	36.45
Step time L (s)	0.82 (0.64–0.91)	0.11	0.31	18.92	53.32	0.73 (0.50–0.86)	0.06	0.16	12.96	34.56	0.93 (0.85–0.97)	0.04	0.12	8.09	22.41
Step time R (s)	0.94 (0.87–0.97)	0.07	0.20	10.84	30.96	0.87 (0.75–0.94)	0.07	0.18	14.46	37.17	0.93 (0.85–0.97)	0.11	0.30	18.78	52.05
Step time MA (s)	0.81 (0.63–0.90)	0.11	0.30	18.37	50.09	0.75 (0.53–0.87)	0.06	0.16	12.72	33.92	0.92 (0.83–0.96)	0.04	0.12	7.96	22.07
Step time LA (s)	0.94 (0.87–0.97)	0.07	0.20	11.14	31.83	0.86 (0.73–0.93)	0.06	0.17	12.62	35.75	0.93 (0.85–0.97)	0.11	0.30	18.93	52.48
Step time symmetry (%)	0.94 (0.87–0.97)	4.40	12.21	35.66	98.84	0.77 (0.75–0.88)	5.27	14.60	57.71	159.97	0.96 (0.92–0.98)	2.67	7.41	27.14	75.22
Step length L (cm)	0.90 (0.79–0.95)	10.17	28.18	18.57	51.46	0.68 (0.42–0.83)	11.35	31.45	17.23	47.75	0.97 (0.93–0.98)	5.35	14.84	8.59	23.82
Step length R (cm)	0.88 (0.76–0.94)	7.51	20.83	12.91	35.80	0.64 (0.37–0.81)	11.00	30.49	16.12	44.69	0.93 (0.84–0.97)	9.12	25.28	14.42	39.96
Step length MA (cm)	0.90 (0.79–0.95)	10.73	29.73	19.44	53.86	0.71 (0.47–0.85)	10.29	28.52	15.45	42.83	0.97 (0.93–0.98)	5.45	15.10	8.71	24.15
Step length LA (cm)	0.87 (0.79–0.95)	7.08	19.63	12.26	33.99	0.61 (0.32–0.79)	11.96	33.16	17.72	49.13	0.93 (0.83–0.97)	9.02	25.00	14.31	39.67
Step length symmetry (%)	0.95 (0.89–0.97)	6.39	17.72	53.08	147.13	0.67 (0.41–0.83)	7.78	21.55	80.71	223.72	0.94 (0.88–0.97)	4.79	13.28	49.77	137.95

*10MWTpref* 10 meter walk test at preferred speed, *10MWTmax* 10 meter walk test at maximum speed, *6MinWT* 6 minute walk tests, *ICC* intraclass correlation coefficient. *95 % CI* 95 % confidence interval, *SEM* standard error of measurement, *SRD* smallest real difference, *SEM%* percentage of the standard error of measurement of the grand mean, *SRD%* percentage of the smallest real difference of the grand mean, *NA* not applicable

The median ICC value calculated from all 21 GAITRite parameter ICC values (Table 3) was 0.93 for the 6MinWT, 0.87 for the 10MWTpref and 0.74 for the 10MWTmax. The ICC values differed between the three tests (*p* <0.001) and were higher for the 6MinWT compared to 10MWTpref and 10MWTmax (for both: *p* < 0.001) and also higher for the 10MWTpref compared to the 10MWTmax (*p* = 0.002). Therefore, the relative reliability of the GAITRite parameters of the three walking tests can be ordered from better to worse as 6MinWT > 10MWTpref > 10MWTmax.

Absolute reliability values of the conventional parameters, expressed as SEM%, of the time needed to cover 10 m were twice as high (10MWTpref: 28.3 %; 10MWTmax: 23.7 %) compared to the walking distance of the 6MinWT (14.1 %, see Table 3). For the GAITRite parameters, SEM% values varied between 4.36 % (extremity-step ratio right leg recorded during the 6MinWT) and 80.71 % (step length symmetry calculated for the 10MWTmax) of the grand mean of the first and second measurement. The median SEM% values calculated from all 21 GAITRite parameter SEM% values (Table 3) were 11.04 for the 6MinWT, 18.88 for the 10MWTpref and 14.59 for the 10MWTmax. In line with the ICC results, SEM% values differed significantly between the tests (Friedman’s test: *p* < 0.001) and were smallest for the 6MinWT compared to 10MWTpref (*p* = 0.001) and 10MWTmax (*p* = 0.001). No significant difference could be found between the 10MWTpref compared to the 10MWTmax (*p* = 0.498). Therefore, absolute reliability of the GAITRite parameters was better for the 6MinWT compared to the two other tests.

## Discussion

The aim of this study was to establish test-retest reliability of the conventional and the GAITRite parameters of the 10MWTpref, the 10MWTmax, and the 6MinWT. Results showed high to very high relative reliability in conventional gait parameters like the time needed to cover 10 m or the distance covered during 6 min. GAITRite parameters recorded during the 6MinWT showed the highest relative and absolute reliability. Second best was the 10MWTpref.

We could not completely confirm our first hypothesis that all the three tests would show good relative reliability. Although the lowest ICC values were still moderate, this is considered not sufficient for tests used to reveal improvement in gait. For the 10MWTmax, only 10 out of 23 parameters showed high or very high relative reliability (ICC ≥ 0.80), while all the parameters of the 10MWTpref and the 6MinWT exceeded this threshold. Concerning our second hypothesis, some parameters showed a low absolute reliability (i.e. considerable measurement errors) between the two sessions. The rather low relative and absolute reliability of the 10MWTmax might be partially explained by its accomplishment. Since we evaluated only the fastest trial, we did not average two or more walks as for the other tests. Averaging several trials reduces variability, which could have increased the reliability. This approach is supported by the observation that the 6MinWT shows the greatest relative and absolute reliability, as there was a mean of 12.67 (SD 4.86, Range 1–22) walks used for analysis in session 1, and a mean of 12.56 (SD 5.64, Range 1–27) walks in session 2.

The results of the reliability of conventional gait parameters are partially consistent with the findings of other studies. Compared to results obtained in children with CP by Thompson et al. [6] the present study revealed higher ICCs and smaller SEM and SRD for the parameter time during the 10MWTmax. In the study of Thompson et al., a period of up to 4 weeks lay between the time-points of measurement [6], which could have led to a more variable performance of the participants between the sessions. Despite that the participants performed the 10MWTmax 1.5 s faster at the second time point (3.1 s faster in a subgroup with GMFCS level III), this change was not significantly different [6]. For the walking distance (6MinWT), however, relative and especially absolute reliability was reported to be considerably better than the results found in our study [6, 8]. In the study by Maher et al., the time between the two 6MinWT measurements lasted only 30 min [8]. Retesting within such a short interval might increase reliability due to the dependence of the measurements. Also in the population of children with myelomeningocele, walking distance appeared more reliable compared to our findings [7]. These children were retested two weeks later and therewith the protocol was more comparable to our study [7]. An important difference that might have led to better results in the study by Maher et al. is the higher level of standardisation: the same tester administered the tests at the same time of day [7]. On the contrary, in our study, we wanted to resemble the clinical situation and abdicated on purpose on a high level of standardisation. In the population of children with cystic fibrosis comparable results of agreements were observed, as the bias (i.e. the average difference between the first and second measurement) was −15.9 m and the limits of agreement were 100.9 m and −132.9 m (Bland-Altman plot) [29].

The results of the GAITRite parameters of the present study are comparable to the results obtained in the study by Wondra et al. [11], where 80 % of the parameters met the ICC threshold of ≥ 0.80 (single and multiple trials, barefoot and with shoes and orthosis) in children with motor disabilities. Considering heterogeneity between the participants, the samples of their and our study are quite comparable. As ICCs depend on the between-subject variance (i.e. a larger between-subject variance leads to greater ICCs), ICCs are usually high in heterogeneous patient groups. This fact has to be considered when discussing these results. Nevertheless, while Sorsdahl et al. [16] investigated test-retest reliability of gait parameters in a rather homogenous group of children with CP (GMFCS levels I and II), they also found (very) high relative reliability, except for the parameter step width, which was not evaluated in our study. The study by Morrison et al. [15] investigating children with developmental coordination disorder. Here, the authors concluded that the wide range of ICC values they obtained could be explained by the variable and inconsistent gait pattern of these children, which could have resulted in low ICC scores [14].

As previously stated, also our second hypothesis that absolute reliability will show considerable measurement errors could be confirmed. While SEM values for the parameter cadence were comparable to the results obtained by Wondra et al. [11], the SEM values for the parameter velocity were larger in our study. The time window between the testing might explain this. Wondra et al. performed their tests on one day, which reduces e.g. the influence of the participant’s day’s form, and, therefore, might lead to more reliable performance [11].

One important aim of this study was to investigate the reliability of the step time- and step length-symmetry, as possible parameters to quantify the quality of gait. While the average symmetry values were quite comparable between the three tests (table 2), the ICC values appeared only excellent for the 10MWTpref and 6MinWT. Sorsdahl et al. [15] also determined the relative reliability of the asymmetry of the step length and found an ICC of 0.82. Their and our test procedures were different: the participants walked barefoot and without their orthosis at self-selected, slow and fast speed, a total of eight walks over a 5.88 m GAITRite walkway. Start and end were 1.5 m before and after the walkway. In the current study, ICC values were better for the 6MinWT and 10MWTpref, but poorer for the 10MWTmax. Symmetry indices were also evaluated in adult patients with stroke [30]. In that study, a step length asymmetry ratio was calculated. Despite differences in calculation, they reported similar ICC values for step length symmetry (ICC 0.81 for one walk, 0.92 for six walks) [30].

Nevertheless, absolute reliability values proved to be utterly poor. SRD% of step length symmetry exceeded 100 % in all three tests, for step time symmetry in the 10MWTmax. We conclude, therefore, that these symmetry parameters appeared promising when considering the quantification of gait quality but, unfortunately, they do not appear reliable enough for longitudinal evaluation.

### Methodological considerations

The heterogeneity of children included in this study reflects the population of children with gait impairments in paediatric neuro-rehabilitation, which is important to determine the generalizability of our results. If these assessments are used for research purposes the examination of reliability in specific populations is needed, [31] since reliability is described as a varying feature and depends on the tested population [24].

Interestingly, the participants walked on average substantially faster during the 6MinWT compared to the 10MWTpref. We hypothesise that this observation is caused by the test instructions. While, for the 10MWTpref, the children were instructed to walk at a self-selected comfortable speed, they had to cover as much distance as possible during the 6MinWT. Apparently, the children were able to walk at such a higher-than-comfortable speed for 6 min.

We assume that the quality of gait of the most severely affected youths was overestimated by the GAITRite, as, for those with poor walking ability, data of the walk required considerable editing with the GAITRite software. By deleting unclear steps, the quality of the walk improved. Editing might also have introduced a higher susceptibility to a bias of the investigator due to unclear decisions on when and how to edit data. Despite that different people edited the walks, they all performed this according to internally formulated guidelines. However, as each walking pattern has its specific characteristics that cannot be described in such guidelines, editing remains to a certain extent subjective. This might have impacted our results (but also those of other GAITRite studies).

Younger children and those with reduced cognitive abilities, although able to follow test instructions, showed quite large differences between the first and second session. This bias might be largely due to a lack of motivation. Motivational aspects have to be kept in mind since they might strongly affect the reliability of any assessment.

ICCs are largely influenced by between-subject variability, i.e. a high ICC does not necessarily also reflect a high absolute reliability. See for example Fig. 2b and f where heterogeneous distributions of absolute step length symmetry indices of 10MWTpref and 6MinWT result in excellent ICC values despite poor absolute reliability values. In clinical and research practice, it is very difficult to make judgements on improvements in individual patients when you have only information about relative reliability. Therefore, we deemed it necessary to investigate also the absolute reliability, i.e. measurement errors. An SRD value informs you what change a patient should achieve before this could be considered a true change, i.e. above chance. Compared to other studies (for example Wondra et al. [11]), we chose a relatively conservative way of calculating the SEM and SRD values, because we included systematic bias, which is not always done.

### Limitations of the study

There are limitations that have to be mentioned. The sample size of our study was rather small, and the sample was very heterogeneous due to the different diagnoses, severity levels and walking abilities. A heterogeneous sample involves large between-subject variance, which results in high relative reliability. Nevertheless, as we wanted to picture reliability of a clinical setting, we decided to keep the sample as heterogeneous as it was. One advantage of a heterogeneous sample is that results can be generalised to a broader population.

The participants were wearing the same orthosis and used the same walking aids for both measurements. Furthermore, we also tried to schedule the measurement time-points at the same time of the day. Since our study was conducted parallel to the rehabilitation programme, this was not always possible. Other factors such as medication, daily activity, and others that we did not control for, might have influenced reliability to a certain extent.

### Clinical implications

A few practical issues have to be mentioned when using the GAITRite walkway system. Firstly, small children might not be heavy enough for the walkway [12]. In our experience, GAITRite measurements are difficult with children with a bodyweight of less than 15 kg since there is not enough pressure on the sensors and the GAITRite does not recognize that there is still a walk in progress. The recording of the walk will stop before it is finished. Secondly, editing difficult walks should be standardised by formulating standardised guidelines, so all walks are edited the same way. Thirdly, automatic and manual editing errors occur. Some are obvious (e.g. calculations are done with 7 steps instead of 8 that are shown in the walk); however, the number of non-recognizable errors is difficult to estimate.

To get a reliable result, several repetitions are recommended, and this can be applied to any test. Nevertheless, the motivational factor and the compliance of the person doing the test must be considered. Compliance decreases with the increasing number of trials, especially in the paediatric field. This might influence reliability to a large extent.

Finally, for clinical purposes, we do not recommend the repeated use of the GAITRite walkway during the 6MinWT because due to a large number of walks, the time required for editing and analysing is considerable.

## Conclusions

The timed walking tests and the simultaneously measured temporo-spatial gait parameters showed moderate to very high relative reliability in all the three timed walking tests. In general, absolute reliability was rather low. Parameters recorded during the 10MWTmax showed lowest relative reliability. Symmetry indices are difficult to use for evaluation purposes, because of poor absolute reliability. The use of the GAITRite system in a paediatric neurorehabilitation setting has its advantages but also disadvantages, as that gait quality of smaller children and those with more severely affected walking ability is likely to become overestimated because unclear steps have to be removed from the analysis.

## Abbreviations

10MWTmax: 10-meter walk test at maximum speed
10MWTpref: 10-meter walk test at preferred speed
6MinWT: 6-minute walk test
95 % CI: 95 % confidence interval
CNS: central nervous system
CP: cerebral palsy
GC: gait cycle
GMFCS: gross motor function classification system
ICC: intraclass correlation coefficient
ICF: International Classification of Functioning, Disability and Health
L: left (leg)
LA: less affected
MA: more affected
MD: median of differences
MMT: manual muscle test
NA: not applicable
R: right (leg)
SD: standard deviation
SEM: standard error of measurement
SEM%: percentage of standard error of measurement of the grand mean
SRD: smallest real difference
SRD%: percentage of smallest real difference of the grand mean
WHO: World Health Organisation
